# Adipose-derived stem cells contribute to cardiovascular remodeling

**DOI:** 10.18632/aging.102491

**Published:** 2019-12-04

**Authors:** Hui Ni, Yiming Zhao, Yongli Ji, Jian Shen, Meixiang Xiang, Yao Xie

**Affiliations:** 1 Department of Cardiology, The Second Affiliated Hospital, Zhejiang University School of Medicine, Hangzhou, China; 2 Department of Endocrinology, The Second Affiliated Hospital, Zhejiang University School of Medicine, Hangzhou, China

**Keywords:** adipose-derived stem cells, vascular remodeling, smooth muscle cell, endothelial cell

## Abstract

Obesity is an independent risk factor for cardiovascular disease. Adipose tissue was initially thought to be involved in metabolism through paracrine. Recent researches discovered mesenchymal stem cells inside adipose tissue which could differentiate into vascular lineages *in vitro* and *in vivo*, participating vascular remodeling. However, there were few researches focusing on distinct characteristics and functions of adipose-derived stem cells (ADSCs) from different regions. This is the first comprehensive review demonstrating the variances of ADSCs from the perspective of their origins.

## INTRODUCTION

Cardiovascular disease contributes to major cause of mortality. Obesity, as a global pandemic, has been one of the main independent risk factors of cardiovascular disease such as hypertension, coronary heart disease and heart failure [1, 2]. When the amount of fat exceeds the capacity of subcutaneous storage, obesity spontaneously occurs [3]. Adipose tissue was initially related to metabolism, where stem cells had been recently identified. These so-called ADSCs displayed the ability of differentiation to vascular lineages such as smooth muscle cells (SMCs), endothelial cells (ECs) and even cardiomyocytes. Therefore, the interaction between ADSCs and vascular remodeling becomes a research hotspot.

Since the discovery of bone marrow mesenchymal stem cells (MSCs), researchers keep trying to search for other stem cell pools. Perfect pools should possess similar therapeutic potential with relatively easy harvest in large quantities and minimally invasive procedure. Under such circumstances, ADSCs were first isolated from the processed lipoaspirate by Zuk and colleagues in 2001 [4]. It reported that adipose tissue contained 500 times more stem cells than the same amount of bone marrow [5, 6]. In addition, ADSCs are less ethically or morally constrained. In addition to subcutaneous region, which is the most common site for adipose-related experiments, ADSCs can also be acquired from visceral, perivascular and pericardial regions. ADSCs, similar to other MSCs such as bone marrow-derived MSCs, express common markers of MSCs and have self-renewal ability [7]. They can also differentiate toward a variety of cell lineages such as adipocytes, osteocytes, chondrocytes and myogenic cells in response to specific culture media. Furthermore, ADSCs could give rise to cardiomyocytes, ECs, SMCs, hepatocytes, epithelial cells, and neural lineage cells [8, 9]. Detailed differentiation capacities of ADSCs toward different cell lineages are listed in Table 1.

**Table 1:** Differentiation abilities of ADSCs into multilineage cells.

**Cell lineage**	**Author**	**Cell source**	**Surface marker**	
			**positive**	**negative**
Adipocytes/ Osteoblasts/ Chondrocytes	Li et al. [99]	Human subcutaneous adipose tissue	CD13, CD29, CD90, CD105, HLA-ABC	CD14, CD19, CD34, CD45, HLA-DR
	Viero Nora et al. [100]	Murine inguinal adipose tissue	CD29, CD44, CD49e	CD11b, CD34, CD45, CD90.2, CD117
	Saler et al. [101]	Human subcutaneous adipose tissue	CD13, CD73, CD90, CD105	CD14, CD34, CD45
	Griffin et al. [102]	Human abdominal subcutaneous adipose tissue	CD73, CD90, CD105	CD14, CD19, CD34, CD45, HLA-DR
Skeletal myocytes	J.-H. Lee and D.M. Kemp [103]	Human subcutaneous adipose tissue	CD13, CD44, CD73, CD90, HLA-ABC	CD34, CD45, CD56, CD184, HLA-DP, HLA-DQ, HLA-DR
	Bayati et al. [104]	Rat gonadal adipose tissue	CD44, CD73, CD90	CD45
Cardiomyocytes	Choi et al. [105]	Human subcutaneous adipose tissue	CD73, CD90, CD105	CD34, CD45
	Chang et al. [106]	Rat subcutaneous adipose tissue	CD13, CD29, CD44, CD90	CD31, CD34, CD45, CD117, CD140a, Flk-1
	Kim et al. [107]	Human intra-abdominal adipose tissue	CD13, CD29, CD44, CD90, CD166, HLA-ABC	CD31, CD34, CD45, CD117, HLA-DR
Smooth muscle myocytes	Parvizi et al. [61]	Human subcutaneous adipose tissue	CD29, CD44, CD90, CD105	CD31, CD45
Endothelial cells	Planat-Benard et al. [31]	Human subcutaneous adipose tissue	CD13, CD34, HLA-ABC	CD14, CD31, CD45, CD144
	Moon et al. [34]	Human subcutaneous adipose tissue	CD34, CD44, CD90	CD31, CD45, Flk-1
	Zhang et al. [37]	Human periumbilical subcutaneous adipose tissue	CD13, CD29, CD44, CD73, CD90, CD105	CD31, CD45
Nerve cells	Kang et al. [108]	Rhesus subcutaneous adipose tissue	CD13, CD59, CD90, HLA-1	CD3, CD4, CD8, CD34, CD45
	Krampera et al. [109]	Human abdominal subcutaneous adipose tissue	CD44, CD73, CD90, CD105	CD11c, CD14, CD31, CD34, CD45, CD123
	Ying et al. [110]	Rat subcutaneous adipose tissue	CD44, CD90	CD34, CD45
Hepatocytes	Lue et al. [111]	Human subcutaneous adipose tissue	CD34, CD90, CD105, CD133	CD13, CD45
	Banas et al. [112]	Human abdominal subcutaneous adipose tissue	CD10, CD13, CD29, CD34, CD44, CD49d, CD59, CD71, CD90, CD105, CD120a, CD124, CD166, SH3	CD11b, CD45, CD48, CD135
Epithelial cells	Brzoska et al. [113]	Human subcutaneous adipose tissue	CD10, CD13, CD44, CD90, vimentin	CD31, CD34, CD45, vWF
Pancreatic islet-like cells	Dhanasekaran et al. [114]	Human omentum adipose tissue	CD73, CD90, CD105	CD31, CD34, CD45, HLA-DR

ADSCs are separated from stromal-vascular fraction (SVF). SVF is the main component in the adipose tissue apart from adipocytes. Currently, isolation of SVF from adipose tissue is mainly by collagenase digestion followed by centrifugation [10]. SVF is composed of heterogeneous cellular population including ECs, SMCs, fibroblasts, pericytes, immune cells, MSCs and other undefined cells [11]. Flow cytometry was applied in plenty of studies to illustrate the CD (cluster of differentiation) antigenic profile of both cultured and freshly-isolated ADSCs [12, 13]. There was common consensus that cultured ADSCs were positive for CD29 [14–17], CD44 [14, 17], CD73 [18, 19], CD90 [14–18] and CD105 [14–18], negative for CD45 [14, 16] and CD31 [14, 15, 18]. The expression of some cell surface proteins remained controversial. For instance, CD34 was expressed in the SVF cells and freshly isolated ADSCs while its expression disappeared after several passages [19, 20].

There are two types of adipose tissue in mammals, white and brown adipose tissue. White adipocyte functions as an energy storage pool, whereas brown adipocyte usually oxidizes fatty acids with specific expression of uncoupling protein-1 [21]. Most studies focus on ADSCs from white fat. Recently, two groups of scientists demonstrated a population of ADSCs from human brown adipose tissue [22, 23]. Silva et al found that ADSCs from adult mediastinal fat were able to differentiate toward both white and brown adipocytes. In addition, these cells expressed higher level of transmembrane protein 26 (TMEM26) and CD137 than white ADSCs [22]. On the contrary, human fetal brown ADSCs only differentiated into classical brown adipocytes with low expression of CD137. Subcutaneous fat and visceral fat are the two main white adipose tissues. They were structurally and functionally different due to distinct characteristics biologically and functionally [24]. In the aspect of yield, the frequency of SVF cells in omental adipose tissue was significantly higher than that in subcutaneous adipose tissue [25]. In addition, it was noted that ADSCs isolated from each gram of visceral fat had more colony forming units than those from subcutaneous fat, implying that the visceral adipose tissue contained more ADSCs [26]. These differences may attribute to different sources of fat tissue. Not only characteristics of ADSCs vary from region to region, but also in one adipose tissue ADSCs display distinct features from subpopulation to subpopulation. A recent single-cell RNA sequencing article identified three major mesenchymal cell populations in the adipose tissue, which were DPP4^+^, ICAM1^+^ and CD142^+^ ADSCs in visceral adipose tissue of obese mice. Each of them had unique biological properties compared to other two subpopulations [27]. Above data demonstrates that ADSCs from different origins or even different subpopulations present distinct characteristics.

In the present article, we will compare the profiles of ADSCs from multiple origins and further discuss their biological functions in vascular remodeling.

## Subcutaneous adipose-derived stem cells

Subcutaneous ADSCs were one of the most commonly used cells in ADSCs research. Subcutaneous fat makes up about 80% of the whole body fat [28]. Subcutaneous ADSCs can differentiate into ECs *in vitro* [9, 29–31]. CD13^+^CD34^+^ cells from subcutaneous SVF cultured in semisolid medium expressed CD31 and vWF. Further experiment demonstrated that these cells could form vessel-like structure by applying matrigel-plug assay subcutaneously in mice [31]. Similar findings were reported in CD34^+^CD31^-^ [30], Flk-1^+^ [29] and CD31^-^CD34^-^c-kit^-^ [32] subcutaneous ADSCs. Subcutaneous ADSCs also undergo endothelial differentiation *in vivo*. In a rat ischemic hindlimb model, injection of subcutaneous SVF cells could recover vascular supply and regenerate numerous CD31^+^ cells lining vessels [31]. Such effect of subcutaneous ADSCs was also confirmed by Cao et al [33] and Moon et al [34]. However, Nakagami et al claimed that injection of subcutaneous ADSCs did not express CD31 and von Willebrand factor in ischemic tissue in their model [32]. Another report showed that promoters of CD31 and CD144 in ADSCs were still methylated in response to endothelial growth factors, suggesting that ADSCs possessed the limited differentiation ability toward ECs lineage [35, 36]. Therefore, whether subcutaneous ADSCs can form functional endothelial cells remains unclear.

To understand the mechanism of ECs differentiation, Zhang et al confirmed that the inhibition of PI3K pathway reduced expression of CD31 genetically as well as capillary density in subcutaneous ADSCs, while inhibition of MAPK did not have such effect [37]. Consistent results were also observed by Cao et al, indicating that PI3K was a key point for ADSCs differentiation toward ECs [33].

ADSCs could also differentiate into smooth muscle cells [38–40]. Rodriguez et al reported that processed lipoaspirate cells exhibited typical SMCs morphology and upregulated the expression of SMCs markers at both transcriptional and translation levels when cultured in SMCs induction media [39]. Specific vascular SMCs-like ion channels were also identified in ADSCs treated by TGF-β1 [41]. *In vivo*, human ADSCs expressed alpha-smooth muscle actin and could survive for several months in the lower urinary tract of immunodeficient mice [38]. These results indicated that ADSCs could give rise to SMCs both *in vitro* and *in vivo*. However, compared to the ECs, the studies of SMCs differentiation in subcutaneous ADSCs are still limited.

**Figure 1:**
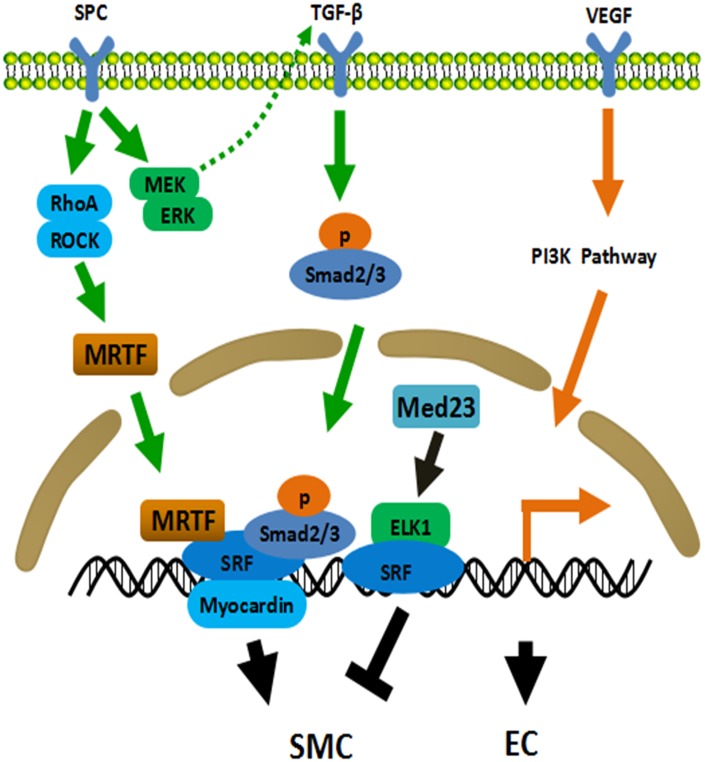
**Mechanisms of ADSCs differentiation into SMC and EC.** TGF-β pathway plays a central role in the differentiation of ADSCs into SMC. ADSCs secrete TGF-β through MEK/ERK-dependent mechanism when treated with SPC. Meanwhile, SPC activates the Rho/ROCK system and subsequently promotes the binding of MRTF and SRF. MRTF, SRF, Smad2/3 and Myocardin jointly initiate the expression of smooth muscle related genes. Med23 represses SMC differentiation via promoting ELK1-SRF to combine with the promoter. PI3K pathway involves in EC differentiation of ADSCs. EC: endothelial cell, SMC: smooth muscle cell, ROCK: Rho-associated protein kinase, SRF: serum response factor, MRTF: myocardin related transcription factor, ELK1: ETS Like-1 protein.

In terms of SMCs differentiation from ADSCs, various potential mechanisms behind cell differentiation were reported [42]. TGF-β signaling pathway was one of them. Many downstream proteins were involved including mitogen activated protein kinase (MEK)/extracellular signal regulated kinase (ERK), c-Jun N-terminal kinase (JNK) and Smad2/3 [42]. Sphingosylphosphorylcholine (SPC) and sphingosine-1-phosphate were also referred to induce the differentiation of ADSCs into SMCs-like cells through G-protein coupled receptor by activating MEK/ERK signalling cascades [43], ras homolog gene family member A (RhoA)/Rho kinase mechanism [44] and TGF-β1 pathway [43, 45]. Autocrine TGF-β1/Smad2 pathway was activated in ADSCs in response to angiotensin-II when differentiated into contractile smooth muscle-like cells [46]. The activation of Notch pathway was also identified [47]. Additionally, bone morphogenetic protein-4 [48] and Med23 [49] were strongly related with the initiation of SMCs differentiation. Deleting Med23 in ADSCs increased SMCs marker myosin regulatory light polypeptide 9 (Myl9). Activin A was indispensable in the early stages of ADSCs differentiation into vascular SMCs when cultured with ECs [50]. However, induction of activin A secretion after stimulating ADSCs towards SMCs diminished ADSCs’ vasculogenic activity [51]. The summary of mechanisms of ADSCs differentiation into SMCs is displayed in Figure 1.

ADSCs can differentiate to cardiomyocytes as well. Planat-Bénard et al obtained functional cardiomyocyte-like cells from ADSCs [52], which were subsequently applied in models of chronic myocardial infarction in rats [53] and nonhuman primates [54], significantly improving heart function. In addition, ADSCs were believed to possess therapeutic effect in acute myocardial infarction [55] and chronic heart failure [56]. Angiogenesis was commonly observed in these disease models facilitated by a series of paracrine factors secreted from ADSCs [57]. miRNAs derived from ADSCs were found to be involved in the process of neovascularization [58].

ADSCs have a great potential in vascular tissue engineering based on their multiple differentiation abilities. A small artificial blood vessel was successfully manufactured by seeding SMCs-differentiated ADSCs on the scaffold [59]. After the application of pulsatile stimulation, the collagen content and biomechanical indicators of artificial vascular wall were significantly improved. Nevertheless, only the medial layer of blood vessels was produced. The feasibility and safety of these vascular grafts also required further investigation. Another research group constructed a two-layered small-diameter blood vessel by applying SMCs and ECs both differentiated from ADSCs [60]. Mechanical stimulation enhanced SMCs generation from ADSCs, which provided a novel method in vascular tissue engineering [61]. ADSCs from different donors varied from on the differentiation and migration of SMCs in tissue engineered blood vessel construction. Diabetic donors showed impaired differentiation abilities of ADSCs while different gender or body-mass index had no such effect [62]. ADSCs from the obese diminished the pro-angiogenic potential owing to reduced expression of miR-126 through inhibition of ERK1/2 MAPK pathway [63]. Another article reported that diabetic ADSCs led to increased inflammation in adipose tissue [64], which may impair vascular remodeling. Besides, ADSCs from the aged failed to facilitate both SMCs differentiation and migration [62]. Cryopreservation of ADSCs also attenuated their differentiation towards SMCs-like cells [65]. Above findings provide new ideas for the application of adipose-derived stem cells in vascular engineering.

ADSCs also have therapeutic effect on abdominal aortic aneurysm treatment via their immunoregulatory capacity, trophic factor production and extracellular matrix synthesis [66]. Xie et al performed tail-vein injection of ADSCs in mice with abdominal aortic aneurysm and found increased aortic FoxP3^+^ regulatory T cells and M2 macrophages, but decreased neutrophils, CD28^−^ T cells and circulating monocytes, suggesting that ADSCs had anti-inflammatory capability. Moreover, they confirmed that the immunosuppression was mainly mediated through paracrine factors secreted by ADSCs [67]. Despite limited application in aneurysm, ADSCs will be promising alternatives from the perspective of vascular tissue engineering [68].

## Abdominal (visceral) adipose-derived stem cells

Subcutaneous and visceral ADSCs displayed inherently different potentials in proliferation and differentiation, even though both of them were able to generate specific cells under corresponding culture conditions [69, 70]. For instance, subcutaneous ADSCs were better and more easily differentiated to mature adipocytes than visceral ADSCs *in vitro* [71, 72]. However, no agreement was reached with respect to the trends for proliferation and differentiation since the results between different study groups seemed contradictory [25, 73, 74]. This may result from inconsistent methods they adopted, such as culture conditions, passage number and donor populations. One article pointed out that visceral ADSCs was a more appropriate cell model *in vitro* for the investigation of molecular mechanisms of metabolic disorders such as obesity because visceral adipose tissue was closely associated with metabolic process [69]. Cells from the superficial layer (subcutaneous) had a greater proliferative rate and induced more outgrowth of neurite-like processes than those from the deep layer [75]. Wee Kiat Ong et al comprehensively screened the cell-surface markers of subcutaneous and visceral ADSCs. The expression of two cell-surface markers, CD10 and CD200, were pointed out to be associated with adipogenic capacity [76]. They discovered that CD10 was specifically expressed in subcutaneous ADSCs and CD200 was predominantly expressed in visceral ADSCs, which could be the specific markers for those ADSCs from different locations [76]. Above researches demonstrated that visceral ADSCs and subcutaneous ADSCs shared a lot of similarities but also had many differences.

Madonna et al described that murine visceral ADSCs in methylcellulose medium could spontaneously undergo neovascularization differentiation, forming CD31^+^CD34^+^ tube-like structures [77]. Another group focused on the adipose tissue-derived myogenic cells. They identified a subpopulation of myogenic cells from the rat visceral ADSCs, and discovered coexpressing telomerase and myocardin A with enhanced abilities of proliferation and differentiation. Further experiments confirmed that myocardin A helped maintain the myogenic stemness of visceral ADSCs through the upregulation of telomerase activation and enhancing myogenic gene expression [78]. The differences of subcutaneous, preperitoneal and visceral ADSCs from morbidly obese women were investigated. Visceral ADSCs secreted the highest levels of IL (interleukin)-6 and MCP (monocyte chemoattractant protein)-1, indicating that they had the most pro-inflammatory effect. On the contrary, preperitoneal ADSCs showed less pro-inflammatory features although they were from internal adipose depot [79].

Due to the promising conclusion from *in vitro* experiments, visceral ADSCs were exploited therapeutically. KDR^+^CD34^−^CD31^−^ cells isolated from human visceral adipose tissue and ADSCs from murine adipose tissue were intravenously and intramuscularly respectively injected to the surgical mice with femoral artery ligation [33, 80]. Both groups concluded the enhanced capillary density and Doppler tissue perfusion scores. Others applied visceral ADSCs in acute and chronic animal models of myocardial infarction [81]. They transplanted rat visceral ADSCs intramuscularly into the rat with left anterior descending coronary artery ligation. Left ventricular end-diastolic volume, left ventricular ejection fraction and cardiac output were improved in rats received ADSCs therapy [53, 82]. Although many *in vivo* experiments have been done, the mechanism whereby ADSCs improved cardiac or vascular functions remained poorly understood. Additionally, whether to use raw or purified ADSCs such as c-kit^+^CD34^+^ was still under debate. At last, data from large animals such as the dogs and the primates were limited. Metabolic dysfunction leads to many diseases such as atherosclerosis and diabetes mellitus. Silvana Baglioni et al believed that the metabolic dysfunctions were related to ADSCs. They assessed the abilities of proliferation and differentiation of subcutaneous ADSCs and visceral ADSCs from the perspective of electrophysiological properties and functional activities. Visceral ADSCs showed less membrane potential, capacitance and K^+^-current parameters, as well as less adiponectin secretion and susceptibility to lipolysis. Such differences may contribute to the development of many metabolic-related diseases [24].

## Other adipose-derived stem cells

Most of the literatures paid heavily attention on the identification and function of subcutaneous and visceral ADSCs for their easy access. Only little effort has been carried out on ADSCs from perivascular, cardiac and other regions. Theoretically, adipose tissue could be adjacent to all vessels and organs except for central nerve system. Such adipose tissues anatomically contact the adventitial side of the arteries and organs, which may play a more important role in vascular remodeling. Mihaela Crisan et al firstly confirmed a perivascular origin for mesenchymal stem cells in multiple human organs, such as skeletal muscle, pancreas, adipose tissue, and placenta, with the absence of expression of hematopoietic, endothelial, and myogenic cell markers. They also identified the multilineage potentials including osteogenic, chondrogenic, and adipogenic potentials but did not further study the relationship between these perivascular MSCs and vascular remodeling [83]. G. Lin et al also succeeded to identify ADSCs from perivascular location where they seemed to express both CD34 and smooth muscle actin. This research demonstrated a more precise location of ADSCs within in human adipose tissue by employing immunofluorescence of SSEA (stage-specific embryonic antigen) 1, STRO-1 and OCT (octamer-binding transcription factor)-4. Based on the results gained above, they proposed that ADSCs were either subsets of pericytes or vascular progenitors surrounding around the vessels [84]. Meanwhile, a population of CD34^+^ ADSCs, which expressed pericyte and MSCs markers, was discovered in periendothelial location, participating endothelial stabilization. These CD31^-^CD144^-^CD34^+^ cells localized in pericytic position and the functional analysis revealed that these cells were associated with vascular structures. The effect of vascular stabilization by CD31^-^CD144^-^CD34^+^ cells was achieved by bidirectional paracrine interaction with endothelial cells, such as VEGF, IL-6, IL-8 and MCP-1. In addition, a substantial proliferative response was detected in these cells when treated with FGF, EGF and PDGF-BB which were all produced by endothelial cells, suggesting a potential interaction with endothelial cells in vascular remodeling [85]. Another study revealed the expression of STRO-1, 3G5 and CD146 in MSCs around the perivascular regions of blood vessels in human adipose tissue sections. However, further relationship between MSCs and vascular function was not discussed [86]. Investigation into adipocyte progenitors revealed that preadipocytes localized to pericytes and endothelial cells of the blood vessels within adipose tissue which shed light on a developmental relationship between these cells [87]. Single-cell transcriptional analysis identified four mesenchymal stem-like cells populations locating in the adipose tissue of the perivascular niche. However, they did not investigate the effect of vascular remodeling for each subpopulation [88]. Overall, the existence of perivascular adipose tissue-derived stem cells has been confirmed but their functions and properties remain unknown where many scientists are currently paying more and more attention.

Cardiac adipose-derived stem cells, another novel type of stem cells separated from adipose tissue surrounding the heart, have been uncovered to be conducive to cardiovascular remodeling over recent years. In accordance to two different adipose depots, cardiac adipose-derived stem cells can be divided into epicardial and pericardial ADSCs. In comparison with adipose stem cells from other sources, cardiac ADSCs were prone to differentiate prominently into cardiovascular cells [89]. What is more, it was known that epicardial ADSCs embraced higher cardiomyogenic potential than pericardial ADSCs [90]. Of note, electrical and mechanical stimulation could strengthen the expression of several specific genes that were worthwhile for cardiodifferentiation [91, 92]. Up to now, it has been continuously reported that cardiac ADSCs exerted advantageous effect on cardiac function improvement and angiogenesis in experimental animal models of myocardial infarction. There was a research showing that perivascular ADSCs provided more potent cardiac reparative activity than subcutaneous ADSCs in view of their intrinsic properties toward myogenic differentiation and vasculogenesis [93]. Furthermore, injury-induced perivascular ADSCs might promote this process as a result of extra HGF production [94]. Angiogenesis is essential for cardiac repair in ischemic heart disease. Epicardial ADSCs isolated from samples within cardiovascular risk factors such as obese, hyperlipidemic and type-2 diabetic obviously impeded vessel formation [95], reminding us that selection of suitable tissue was vital for cell therapies. Differentiation and maturation of the transplanted cells collaborated with more relevant paracrine effect were considered to account for angiogenesis [96]. Agreed with this mechanistic basis, Wang et al speculated that newly formed cardiomyocytes might be partially derived from self-replicating cardiac cells albeit the instructive signals between ADSCs and cardiomyocytes remained unclear [97]. Taken together, cardiac ADSCs represent promising candidates for future use in cardiovascular regeneration therapies even though these cells are not easily accessible [98].

Adipose-derived stem cells around other regions, despite being rarely discussed, are of great value, which calls for extensive and in-depth studies to explore their physiological and pathological mechanisms.

## Summary

Stem cell therapy presents a promising future in the field of regenerative medicine. Generating a large number of cardiovascular cell lineages is a key step in cell therapy of cardiovascular disease. ADSCs are among one of the most promising cell types for translational medicine and provide unprecedented opportunities for their easier isolation and less ethical aspects. In addition to differentiation into osteoblasts, cartilage and fat cells, ADSCs have been shown to differentiate into endothelial cells, smooth muscle cells and cardiomyocytes. The latter three are closely related to cardiovascular remodeling and disease progression. Previous studies demonstrated that different sources of ADSCs have different proliferative and differentiation abilities. In this review, we roughly divided ADSCs into subcutaneous, visceral and other ADSCs based on their origins. The properties of three different ADSCs were carefully compared to each other. This is also the first review of the relationship between cardiovascular remodeling and ADSCs from different origins. Although there are still many obstacles to overcome, we hope that clinical application of ADSCs will be widely used in the near future.
